# Inflammation in ST- elevation myocardial infarction: risk factors, patterns of presentation and association with clinical picture and outcome, an observational study conducted at the Institute of Cardiology-National Hospital of Sri Lanka

**DOI:** 10.1186/s12872-019-1104-5

**Published:** 2019-05-14

**Authors:** Mitrakrishnan Rayno Navinan, Sepalika Mendis, Sumudu Wickramasinghe, Ambiga Kathirgamanathan, Tharanga Fernando, Jevon Yudhisdran

**Affiliations:** 1 Institute of Cardiology, National Hospital of Sri Lanka, Colombo, Sri Lanka; 2 District General Hospital Killinochi, Colombo, Sri Lanka

**Keywords:** Ischaemic heart disease, Inflammation, CRP, Fever, STEMI

## Abstract

**Background:**

Inflammation in myocardial infarction has a complex immunogenic origin and is suspected to be closely involved in its aetio-pathogenesis as well as outcome. In this study the objective was to further elucidate the clinical correlations of inflammation using clinical parameters and basic inflammatory markers and how it correlates with patient risk parameters, imaging findings and outcome.

**Methods:**

An observational descriptive cross sectional study was carried out at the Institute of Cardiology, National Hospital of Sri Lanka, where consenting patients presenting for further management of ST- elevation myocardial infarction were recruited. Venous blood samples were collected on admission to assess C-reactive protein levels and on a timed manner to asses Troponin I levels as well as on subsequent days to performs whole blood analysis. Patients underwent 6 hourly axillary temperature assessment. All patients underwent 2D transthoracic echocardiographic analysis via biplane Simpson’s method to ascertain ejection fraction as well.

**Results:**

Eighty eight subjects were recruited into the study. Fever was noted in 20.5% (*n* = 18). Fever was usually intermittent and seen commonly between day 1 and 3 post-acute myocardial infarction. Haematological abnormalities indicative of inflammation were also observed as whole blood analysis demonstrated predominant leukocytosis and elevated C-reactive protein levels. Significant correlation was noted between presence of leukocytosis (*P* = 0.033) and fever as well as with the presence of diabetes mellitus (*P* = 0.005). Development of acute heart failure also showed significant correlation with leukocytosis (*P* = 0.002). Correlation was also observed between LV dysfunction and elevated C-reactive protein and Troponin I levels with *P* values of *P* = 0.023 and *P* = 0.011 (*P* < 0.05) respectively.

**Conclusions:**

Inflammation is appreciated following acute myocardial infarction. Biochemical evidence of inflammation is commonly seen. Clinical manifestation as fever however is seen less often. Patient factors correlate poorly with inflammation but diabetes mellitus may have a contributory role. Whole blood analysis derangement is a simple test that correlates well with inflammation as well as presence of fever and development of heart failure. Inflammation also correlated with left ventricular dysfunction and may thus have an impact on clinical morbidity and mortality. Delineating associates of inflammation will hopefully help improve therapy of myocardial infarction.

## Background

Ischaemic heart disease (IHD) is acknowledged to be the leading cause of mortality and morbidity globally by the World Health Organization, and Sri Lanka is ranked 6th highest among South Asian countries with regards to the number of deaths recorded due to IHD with a mortality of 15.9 per 1000 in the study published by Finegold et al. in 2008 [1]. The trend from 2008 appears to continue as IHD still remains the leading cause of mortality according to available data in 2017 [2]. Though the spectrum of clinical presentation of IHD shows variation, the underlying principle is that of atherosclerosis with plaque formation and rupture. The origin of atherosclerosis formation is multifactorial with well-known risk factors both acquired and hereditary. Attention however has now diverged with greater understanding of immunogenic pathways, regarding the role of inflammation in IHD, which has been questioned and studied. Post mortem analysis of tissue specimen with microscopic analysis along with in vivo studies demonstrate the many facets of inflammation contribute to the aetio-pathogenesis of acute coronary syndrome. Inflammation in myocardial infarction is complex and occurs by multiple pathways and has a bimodal distribution in regard to its origin and resolution according to Ong et al. [3]. According to Mulvihil et al., inflammation acts as a key component both as an aetiological agent in its origin, its evolution by promoting acute plaque rupture, and overall having a negative impact on vessel anatomy and function after the acute insult [4]. The impact of inflammation can even manifest as systemic symptoms. Fever is a form of an immune-pathogenic response of the body to chemical mediators. Since acute coronary syndrome is a complex process involving many mediators which has the propensity to act as pyrogens, it is not surprising that the clinical event of fever has both been observed in acute coronary events and had its clinical significance questioned in regard to future outcome [5] as fever itself can trigger a physiological cardiovascular response such as tachycardia which may be detrimental in the acute setting. Biochemical assays and haematological investigations such as C-reactive protein (CRP) and white blood cell counts with differential counts are commonly available and readily used markers, amongst many other indicators in the clinical setting that represent inflammation and helps to give us an objective numerical representation of a very complex systemic process. Their value in acute coronary events have been questioned and have been proven with large scale studies showing that elevated values correlate with prognosis of patient outcome, both long and short term alike [6]. In this study we hope to elucidate further the clinical correlations of inflammation in a ST-elevation myocardial infarction (STEMI) populace using clinical parameters and basic inflammatory markers and how these correlate with patient risk parameters, imaging findings and outcome.

## Methods

An observational descriptive cross sectional study was carried out at the Institute of Cardiology, National Hospital of Sri Lanka. Data was collected over a period of 6 months from February 2018. All adult patients above the age of 18 years who were taken over for further management of STEMI irrespective of the chosen path of treatment were considered eligible candidates if consenting for the study. Individuals who had been identified through clinical assessment via history & examination to have an identifiable source of sepsis or ongoing inflammation of alternate aetiology e.g., inflammatory or connective tissue disorders were excluded from the study. Patients with non ST elevation MI (NSTEMI), unstable angina (UA) & stable angina were also excluded from the study. Convenience sampling was utilized to enroll patients. Following parameters were collected; patient demographic data, relevant patient medical history, clinical parameters including fever assessment, blood pressure and heart rate. Variables considered in our study include presence or absence of fever, co-morbidities (diabetes mellitus [established or newly diagnosed], hypertension), patient habits (alcohol consumption, smoking), symptomatology (ongoing chest pain) of the patient, complications (acute heart failure), haematological abnormalities favoring inflammation (whole blood analysis, C-reactive protein), myocardial damage (Troponin I titre), time (time to admission and duration of stay), and patient outcome. The objective was to ascertain correlation between these variables and features favoring inflammation. For diabetes mellitus two values of fasting blood sugar >7mmo/L was considered suggestive, normal leukocyte range was considered as 4000–10,000 cell/microliter. Normal reference range of C-RP was considered < 6 mg/dL. Reference range for standard troponin I is < 0.05 ng/dL. Axillary temperature was measured every 6 h. A fever chart was kept for all patients from the time of admission to that of discharge. A calibrated digital thermometer was used to measure temperature. A recorded value above 98.4°F (36.8°C) was considered to be suggestive of presence of fever for this study. Haematological investigations included an initial venous blood sample for C-reactive protein levels, followed later by baseline troponin I levels after 8 h, calculated from the maximal chest pain for a primary PCI and for those taking pharmaco-invasive strategy or on admission for those admitting late. Daily whole blood analysis during the course of stay was done. Fasting blood sugar levels were done on day 2. A newly diagnosed diabetic via elevated fasting blood sugar was confirmed with repeat fasting blood sugar as per standard guideline practice. Additionally electrocardiographic data was collected & transthoracic 2D echocardiographic assessment was done within 24 h of presentation for all patients. Transthoracic 2D echo was done with a Philips IE 33 echo machine. To analyze ejection fraction biplane Simpsons method was utilized. If the patients chose to undergo further management through intervention, coronary angiographic data was also collected. All investigations were done using available laboratory facilities at the National Hospital of Sri Lanka, with no out of pocket expenditure for the patient.

## Results

The number of eligible consenting patients was *n* = 88 in a period of six months. Majority were male *n* = 76, constituting 86.36% of the study population. The minimum age was 24 years and the maximum age was 77 years, with a mean age of 50.19 years (Std. deviation ±13.132). Since the study populace was that of STEMI patients, most 47.7%(*n* = 42) had anterior STEMI, while 37.5% (*n* = 33) had inferior STEMI and 14.8% (*n* = 13) had anterolateral STEMI. Of the study subjects 76.1%, (*n* = 67) presented on the same day of the myocardial infarction(MI), regardless of whether it was for primary percutaneous coronary intervention(PCI) or pharmaco-invasive strategy PCI. *N* = 14 individuals presented on 2nd day of MI. While 4.5% (*n* = 4) and 3.4% (*n* = 3) of the study populace presented on 3rd and 4th day following their acute coronary event respectively. Majority of the patients 54.5%, (*n* = 48) underwent primary PCI, while 44.3% (*n* = 39) of the enrolled subjects underwent pharmaco-invasive strategy. Only *n* = 1 underwent isolated thrombolysis. Of those who underwent coronary angiography, most *n* = 49 (56.32%) had single vessel disease on angiography, of which *n* = 25 had only acute plaque rupture with non-infarct related coronary arteries appearing normal on coronary angiography. Amongst the *n* = 37 (42.52%) who had multi vessel disease, *n* = 20 had dual vessel disease and *n* = 17 had triple vessel disease. One individual had minor coronary artery disease. Overall the mean time of presentation was 1.35 days with a std.deviation of ±0.728 days. The duration of hospitalization varied from a minimum of 2 days to maximum of 15 days, and the mean duration of stay was 3.67 days (±1.59). With regard to risk factors, most 69.3% (*n* = 61) had a history of smoking, of which the mean duration was 9.96 pack years. Only 30.7% (*n* = 27) gave a history of consumption of alcohol. Majority 96.6% (*n* = 85) denied a history of substance abuse. 34.1% (*n* = 30) of the populace were established diabetics, while 58 individuals claimed to be non-diabetics. Of the 58, fifteen (*n* = 15) were diagnosed to be diabetics following their presentation. Most 78.4% (*n* = 69) did not have a history of hypertension. For a majority 92% (*n* = 81) of the individuals this was the first episode of an acute coronary event or ischaemic heart disease. Mortality during our study period was 3.4% (*n* = 3).

Of the 88 individuals, only 20.5% (*n* = 18) developed fever during their period of stay. Of the 18 who were noted to have fever, majority *n* = 14 had fever only for a single day. All 18 had an intermittent pattern of fever. Of the eighteen, 38.89% (*n* = 7) were noted to have fever during day 1 (within 24 h) of MI. While 27.78% (*n* = 5) had fever on day 2 and 33.33% (*n* = 6) had fever on day 3. Only 16.67% (*n* = 3) had fever on 4th day of MI while *n* = 1 (5.56%) had fever on the fifth day following Ml. The highest observed fever was 102°F (38.8°C).

Most 81.8% (*n* = 72) of the patients had ongoing chest pain on takeover. Majority 75% (*n* = 66) had deranged whole blood analysis values, with a predominant leukocytosis (mainly neutrophil count) being an observed phenomenon. Thrombocytosis was not noted. Day 1–4 analysis of white cell blood count derangement is summarized in Table 1.

**Table 1:** Whole blood analysis- White Blood Cell count (WBC) variation Vs. Days

Day-WBC	n	Average Neutrophil %	Minimum	Maximum	Mean	Std. Deviation
Day 1-WBC	82	78.9	6.1	34.0	14.29	4.93
Day 2-WBC	67	75.3	5.58	25.20	12.89	3.99
Day 3-WBC	42	72.9	7.70	22.50	11.98	3.31
Day 4-WBC	10	75.3	9.10	14.90	11.95	1.64

Analysis of C - reactive protein levels revealed that 77.3% (*n* = 68) had elevated values above reference range (< 6 mg/dL). The maximum noted value was 261 mg/dL with a mean of 53.12 mg/dL (± 56.69 mg/dL). Troponin I titres revealed values ranging from 0.54 ng/dL to maximum of 50.0 ng/dL (reference < 0.05 ng/dl) with a mean of 20.25 ng/dL (±12.95 ng/dL). Spearman’s rank correlation (rs) was used to discern for association between Troponin I levels and the presence of fever, elevated leukocytosis, ongoing chest pain, acute heart failure and clinical outcome all of which failed to show a statistically significant correlation. However Troponin level showed a positive correlation with CRP levels and a negative correlation with left ventricular (LV) function [measured by ejection fraction (EF)] with *P* values of 0.016 & 0.011 respectively (*P* < 0.05) Table 2.

**Table 2:** Factors correlating with Troponin I titre levels in the STEMI sample population

Variables	Spearman’s rank correlation coefficient	Significance - 2 tailed
Presence of Fever	0.094	0.386
Elevated WBC levels	0.115	0.287
Elevated CRP levels	0.257	0.016
Acute heart failure	−0.144	0.184
Impaired EF	−0.276	0.011
Ongoing pain	−0.032	0.767
Outcome	0.062	0.565

Clinical parameters suggestive of inflammation, specifically the presence of fever showed statistical significance with regard to deranged white blood cell counts in whole blood analysis, with a Chi square *P* value of 0.033 (< 0.05) and presence of diabetes mellitus 0.005 (< 0.05). But presence of fever did not show any significant relationship with patient factors such as gender, co-morbidities (hypertension), acquired risk factors (smoking, alcohol consumption, recreational drug use), time of admission (early versus [within one day] that of late [after one day]) and elevated C-reactive protein as all had a *P* > 0.05. Table 3.

**Table 3:** Factors correlating with presence of fever in the STEMI sample population

Variable	N	df	Pearson’s Chi-square value	Asymptotic significance (2 sided) (*p* value)
Gender	88	1	1.255	0.263
Presence of diabetes mellitus	88	1	7.778	0.005
Presence of hypertension	88	1	1.843	0.175
History of smoking	88	1	0.090	0.765
History of alcohol consumption	88	1	0.075	0.784
History of recreational drug use	88	1	0.799	0.371
Time of admission (early vs delayed)	88	1	1.117	0.291
Elevated WBC on day 1	88	1	4.563	0.033
Elevated CRP levels	88	1	0.645	0.422

Further analysis was done regarding the association of diabetes mellitus with markers of inflammation (CRP levels & leukocytosis) and LV dysfunction, which revealed statistically significant relationships with elevated CRP levels as well as leukocytosis, with *P* values of 0.040 and 0.044 (*P* < 0.05) respectively Table 4.

**Table 4:** Factors correlating with diabetes mellitus in the STEMI sample population

Variables	N	df	Pearson’s Chi-square value	Asymptotic significance (2 tailed) (*P* value)
Elevated WBC levels	88	1	4.052	0.044
Elevated CRP levels	88	1	4.219	0.040
Impaired EF	88	1	0.437	0.509
Outcome	88	1	0.604	0.437

The time of admission early (within day 1 of MI) compared to that of late (after day 1 of MI) did not show any statistically significant correlation when analyzed with deranged whole blood counts or elevated C-reactive protein levels (*P* > 0.05).

The association of ongoing chest pain, a symptom of ongoing ischaemia was analyzed with inflammatory parameters including presence of fever and deranged whole blood counts but failed to demonstrate statistical significance (*P* > 0.05). However ongoing chest pain correlated significantly with C-reactive protein with a *P* value of 0.039 Table 5. Left ventricular function impairment was assessed using Ejection fraction, which was categorized as either preserved ≥50% or impaired < 50%. When analyzed with presence of fever or elevation of WBC it failed to show any statistical significance (*P* > 0.05). However elevated CRP was significantly associated with cardiac functional impairment with a P value of 0.023 (*P* < 0.05) Table 5. Spearman’s correlation coefficient revealed a negative correlation (rs = − 0.249, *P* = 0.022) between the CRP level and ejection fraction. Nearly 50.6% (*n* = 44) developed clinical features of acute heart failure in our study populace. Development of clinical feature of acute heart failure failed to correlate with presence of fever and elevated CRP (*P* > 0.05), but demonstrated significant correlation with leukocytosis (*P* = 0.002) Table 5.

**Table 5:** Factors correlating with symptom of ongoing chest pain, left ventricular function and acute heart failure in the STEMI sample population

Variable	N	df	Pearson’s Chi-square value	Asymptotic significance (2 sided) (*p* value)
Factors correlating with symptom of ongoing chest pain				
Elevated WBC levels	88	1	1.401	0.237
Elevated CRP levels	88	1	4.256	0.039
Presence of fever during stay	88	1	0.760	0.383
Factors correlating with left ventricular function				
Elevated WBC levels	88	1	0.59	0.430
Elevated CRP levels	88	1	5.20	0.023
Presence of fever during stay	88	1	2.08	0.149
Factors correlating with clinical features of acute heart failure				
Elevated WBC levels	88	2	12.789	0.002
Elevated CRP levels	88	2	3.365	0.132
Presence of fever during stay	88	2	1.151	0.470

Angiographic results which were broadly categorized as acute plaque only or acute on background atherosclerotic change or alternatively categorized as single vessel disease vs. multi vessel disease when analyzed with inflammatory parameters including presence of fever, elevation of WBC and C-reactive protein levels failed to demonstrate statistical significance (*P* > 0.05).

Outcome was analyzed based on duration to discharge as standard versus that of delayed (3 days or less vs. greater than 3 days) and was analyzed whether presence of fever or inflammatory parameters including deranged whole blood counts, or C-reactive protein had an association, but there was no statistical significance *P* > 0.05. Additionally *n* = 2 had developed ventricular septal defects (VSD) & *n* = 44 (50%) developed clinical features of acute heart failure and three patients (*n* = 3) died during their stay. Outcomes were also analyzed by assessing whether patients survived or had poor outcome (e.g., high risk complications-VSD, death) correlated with fever during stay and inflammatory parameters including deranged whole blood counts or C-reactive protein but no significant statistical association was noted (*P* > 0.05).

## Discussion

Myocardial infarction can cause myocardial necrosis, and in the immediate post MI period results in sterile inflammation mediated by endogenous inflammatory mediators through a slew of complex process e.g. reactive Oxygen species, damage associated molecular patterns, inflammatory cells, chemokines and cytokines namely Interleukin 1 etc., [3]. These mediators alter the body temperatures set point in the hypothalamus and induce fever. The affected myocardial territory size and duration affects the amount of mediators produced [7] implying that fever possibly correlates with amount of cardiac involvement. We emphasize fever in our study because it is a simple yet significant imbalance in the human physiology that underscores the presence of inflammation. In our study populace Inflammation was demonstrated both clinically in the form of fever as well as through haematological abnormalities. Though haematological parameters such as leuckocytosis and elevated C-reactive protein levels were abnormal in most of the study populace, fever was not a constant finding as only around 1/5th of the study populace elicited a febrile response. This number is similar to a study by Wang et al. in 2017 who also noted only 16.3% incidence of fever among 276 ST-elevation MI patients [8]. It is interesting to note that most therapy given in acute myocardial infarction acts to suppress fever through many pathways. The antiplatelet agent Aspirin is established and accepted to have an antipyretic effect [9]. Statins have anti-inflammatory effect [10] and even beta blockers contribute toward temperature reduction through both central mediation and peripheral mechanisms [7]. Thus it is reasonable for Kacprazak et al. to state that fever is not a commonly observed symptom in myocardial infarction [11]. The highest fever observed in our study was 102°F (38.8°C), despite high levels of inflammation as observed by CRP and leukocytosis the febrile response had a roof limit, a finding mirrored in a study by Lofmark who also noted a maximum observed fever of 102.2°F (39.0°C) which was also limited by time as well usually lasting less than a week with majority having fever after day 1 and before day 5 [12] which also matched our findings as 14 of the 18 who had fever had it between day 2 and day 5. The pattern of fever we observed was also intermittent. In an analysis by Ong et al., they surmise that reperfusion will also result in injury, anywhere between minutes to hours- naming it late reperfusion injury. This according to them will exacerbate the pro-inflammatory response seen up to 24 h after reperfusion [3]. It is interesting to note that the populace in which fever was noted had a delayed onset of it, and this could be possibly explained by the reperfusion injury phenomena. Ong et al. also postulates the onset of anti-inflammatory period from day 4–7 onwards in which the body goes into reparative phase to negate inflammation [3]. Alternatively acute pericarditis following acute MI is another valid explanation for fever and can usually be seen after day 3. But this classically has other features including a typical pattern of pain different to that of ischaemia, along with suggestive ECG changes [13, 14] none of which were observed in our patients and hence was considered an unlikely aetiology in this study populace. Thus one could surmise that fever following myocardial infarction is due to acute MI or potentiated by reperfusion adding further insult to injury and is limited by time. Accordingly it is reasonable to state that persistence of fever beyond a reasonable frame of time [15] and a suspicious clinical pattern of fever should point towards an alternate diagnosis.

The clinical significance of fever is that its potential correlation with the extent of infarction as suggested by Herlitz et al. who hypothesized that a positive association exists between enzyme-estimated infarct size and body temperature and the persistence of fever may imply the presence of ongoing ischaemia [16]. The symptom of ongoing ischemia did not correlate with the presence of fever in our study populace but was found to correlate with CRP levels suggesting a relationship of symptomatology to inflammation. Our analysis also revealed presence of biochemical & haematological evidence of inflammation in the form of elevated C-reactive protein and leukocytosis in a majority of our study populace. Majority had elevated C-reactive protein but there was no correlation with the occurrence of fever. But interestingly there was correlation of leukocytosis with fever, suggesting that whole blood analysis is a simple test that also easily correlates with the inflammation in STEMI. However our findings contradict Ben-dor et al. findings who found the opposite in his study, where High sensitivity-CRP (Hs-CRP) and not WBC correlated with fever [17]. However a pertinent difference was Hs-CRP was used in that study and in this we used standard CRP. In addition though we analyzed Troponin I levels we could not find a clinical association between the incidence of fever and its levels, as opposed to those documented in literature [17]. We also attempted to analyze patient factors that may contribute towards that of inflammation, including gender, comorbidities e.g., hypertension and acquired risk factors eg., smoking & alcohol consumption, none of which showed any association, possibly suggesting that inflammation may be independent of these factors. But diabetes mellitus showed a statistical association with leukocytosis as well as elevated CRP levels. Heo et al. [18] in their study also compared the role diabetes had to play in different sub-types of ischaemic heart disease and concluded that diabetic STEMI populace had an overall elevated CRP level and this was a significant association compared to the non-diabetic populace, suggesting diabetes as a potential contributory agent in inflammation in the STEMI populace.

Transthoracic 2D echo was utilized to assess cardiac function through biplane Simpson’s method. Focused 2D echo assessment was done within the first day of admission in all patients admitted for further management. Amongst our study populace LV function showed an inverse correlation with C-reactive protein levels and levels of Troponin I elevation. In Naito et al. study left ventricular pump failure, LV enlargement and low EF correlated with presence of fever [19]. Additionally, a study by Azai et al. revealed LV based complications e.g. aneurysmal LV and rupture of LV wall was noted at a higher incidence when inflammation was noted with high CRP [20], thus suggesting that inflammation correlates with LV function and complications which translates into increased morbidity and mortality. Half the populace in our study developed clinical features of acute heart failure, and when analyzed against markers suggestive of inflammation showed significant correlation with the presence of leukocytosis. This finding is in keeping with the observations made by Barron et al. as well as Jan et al. [21, 22], favoring the hypothesis that inflammation potentially correlates with poor outcome. In our study populace though we observed a few deaths & other high risk complications such as VSD, and analysis of inflammatory parameters clinical, biochemical or hematological markers failed to show a statistical significance (*P* > 0.05). This negative findings may be attributable to the low power of our study. However fever is noted to be an independent risk factor that has prognostic significance and may determine adverse outcomes both in the form of negative cardiac remodeling and cardiac events [19] and animal model studies have demonstrated temperature may correlate with infarct size, and interestingly elevation of body temperature worsens infarct size as well [23, 24]. Furthermore successful reperfusion via intervention can also be affected negatively when inflammation is noted with high CRP [25]. This implies that active detection of fever and markers of inflammation, a clinically simple task, will help us potentially anticipate complications in patients who are already a high risk group.

Our study would have benefitted from use of alternate investigations such as high sensitivity CRP and more frequent monitoring. Since the study was done using available resources within the National Hospital system based on free health care we utilized available standard investigations at our disposal. LV dysfunction measured by global strain analysis would have yielded more data in addition to Simpsons EF. Additionally a higher powered study with long term follow up would have given more insight as to future implications and consequences of inflammation following acute MI.

Furthermore considering the different spectrum of presentation of type 1 acute myocardial infarction, extending the inclusion criteria to recruit non-STEMI patients into the study would have been an interesting option. However, though both non-STEMI and STEMI belong to type 1 universal definition of myocardial infarction and have a common underlying basis of origin of atherosclerosis, based on the way of presentation and observed patterns of recurrence in patient populations, non-STEMI and STEMI are hypothesized to be two different entities on a molecular level [26]. Furthermore studies have also shown that levels and pattern of inflammation vary between Non-STEMI and STEMI populations [27]. In this study we decided to focus on STEMI patients alone to ensure uniformity in analysis. That being said, a larger study both in number and spectrum (UA, NSTEMI, STEMI) may possibly yield interesting data and outcomes.

The role of modulating inflammation as a form of active treatment has already been questioned and trialed and with mounting evidence it is now assumed that rather than targeting one single pathway, simultaneously inhibiting multiple avenues of inflammation possibly holds promise for further enhancing management of myocardial infarction [3]. Thus having better understanding of inflammation in myocardial infarction and its correlates will no doubt be useful for future therapy.

## Conclusions

Inflammation is seen and appreciated in ST-elevated myocardial infarction both clinically and through biochemical abnormalities. Clinical pattern of fever is usually demonstrable after day one and maybe associated with reperfusion injury as well. Fever is limited by intensity and duration in the natural course of ST-elevation myocardial infarction. Whole blood analysis demonstrating leukocytosis is a simple test that correlates well with inflammation in acute STEMI especially in those where fever was observed and also correlates with development of clinically demonstrable heart failure. Inflammation and its indicators seem independent of patient dependent factors such as level of coronary involvement, acquired risk factors and habits but diabetes mellitus may have a contributory role towards inflammation in STEMI as demonstrated by its significant relationship observed with CRP levels and leukocytosis. Inflammation tends to correlate with cardiac morbidity as LV dysfunction was noted to correlate significantly with that of inflammatory marker CRP. Inflammatory markers such as CRP and leukocytosis can correlate with clinical morbidity and may indicate poor outcomes. A higher powered study and use of high sensitivity CRP and more frequent analysis and longer follow up may reveal more detail.

## Abbreviations

CRP: C-reactive protein
EF: Ejection fraction
Hs-CRP: High sensitivity C reactive protein
IHD: Ischaemic heart disease
MI: Myocardial infarction
NSTEMI: Non ST elevation MI
PCI: Percutaneous coronary intervention
STEMI: ST-elevation myocardial infarction
UA: Unstable angina
VSD: Ventricular septal defects
WBC: White blood cell

## References

[1] Finegold JA, Asaria P, Francis DP (2013). Mortality from ischaemic heart disease by country, region, and age: Statistics from World Health Organisation and United Nations. Int J Cardiol.

[2] Washington Uo. IHME-Sri Lanka: University of Washington; 2017 [cited 2018 20/11/2018]. Institute for Health Metrics and Evaluation]. Available from: http://www.healthdata.org/sri-lanka.

[3] Ong SB, Hernandez-Resendiz S, Crespo-Avilan GE, Mukhametshina RT, Kwek XY, Cabrera-Fuentes HA (2018). Inflammation following acute myocardial infarction: multiple players, dynamic roles, and novel therapeutic opportunities. Pharmacol Ther.

[4] Mulvihill NT, Foley JB (2002). Inflammation in acute coronary syndromes. Heart..

[5] Zaman S, Ahmed S (1988). Temperature rise in patients with acute myocardial infarction. JAMC..

[6] Blake GJ, Ridker PM (2003). C-reactive protein and other inflammatory risk markers in acute coronary syndromes. J Am Coll Cardiol.

[7] Risoe C, Kirkeby OJ, Grottum P, Sederholm M, Kjekshus JK (1987). Fever after acute myocardial infarction in patients treated with intravenous timolol or placebo. Br Heart J.

[8] Jang WJ, Yang JH, Song YB, Chun WJ, Oh JH, Park YH, et al. Clinical significance of Postinfarct fever in ST-segment elevation myocardial infarction: a cardiac magnetic resonance imaging study. J Am Heart Assoc. 2017;6(4). 10.1161/JAHA.117.005687 PMC5533041 28438740

[9] Aronoff DM, Neilson EG (2001). Antipyretics: mechanisms of action and clinical use in fever suppression. Am J Med.

[10] Antonopoulos AS, Margaritis M, Lee R, Channon K, Antoniades C (2012). Statins as anti-inflammatory agents in Atherogenesis: molecular mechanisms and lessons from the recent clinical trials. Curr Pharm Des.

[11] Kacprzak M, Kidawa M, Zielinska M (2012). Fever in myocardial infarction: is it still common, is it still predictive?. Cardiol J.

[12] Lofmark R, Nordlander R, Orinius E (1978). The temperature course in acute myocardial infarction. Am Heart J.

[13] Gregoratos G (1990). Pericardial involvement in acute myocardial infarction. Cardiol Clin.

[14] Sondhi S, Mahajan K, Mehta A, Dev M (2017). Post MI pericarditis or recurrent infarction-diagnostic dilemma. Open Access Text.

[15] Gibson TC (1974). The significance of fever in acute myocardial infarction: a reappraisal. Am Heart J.

[16] Herlitz J, Bengtson A, Hjalmarson A, Wilhelmsen L (1988). Body temperature in acute myocardial infarction and its relation to early intervention with metoprolol. Int J Cardiol.

[17] Ben-Dor I, Haim M, Rechavia E, Murininkas D, Nahon M, Harell D (2005). Body temperature - a marker of infarct size in the era of early reperfusion. Cardiology..

[18] Heo JM, Park JH, Kim JH, You SH, Kim JS, Ahn C-M (2012). Comparison of inflammatory markers between diabetic and nondiabetic ST segment elevation myocardial infarction. J Cardiol.

[19] Naito K, Anzai T, Yoshikawa T, Maekawa Y, Sugano Y, Kohno T (2007). Increased body temperature after reperfused acute myocardial infarction is associated with adverse left ventricular remodeling. J Card Fail.

[20] Anzai T, Yoshikawa T, Shiraki H, Asakura Y, Akaishi M, Mitamura H (1997). C-reactive protein as a predictor of infarct expansion and cardiac rupture after a first Q-wave acute myocardial infarction. Circulation..

[21] Jan AF, Habib S, Naseeb K, Khatri MA, Zaman KS (2011). High total leukocyte count and heart failure after myocardial infarction. Pakistan Heart J.

[22] Barron HV, Cannon CP, Murphy SA, Braunwald E, Gibson CM (2000). Association between white blood cell count, epicardial blood flow, myocardial perfusion, and clinical outcomes in the setting of acute myocardial infarction: a thrombolysis in myocardial infarction 10 substudy. Circulation..

[23] Chien GL, Wolff RA, Davis RF, van Winkle DM (1994). “Normothermic range” temperature affects myocardial infarct size. Cardiovasc Res.

[24] Duncker DJ, Klassen CL, Ishibashi Y, Herrlinger SH, Pavek TJ, Bache RJ (1996). Effect of temperature on myocardial infarction in swine. Am J Phys.

[25] Dibra A, Mehilli J, Schwaiger M, Schuhlen H, Bollwein H, Braun S (2003). Predictive value of basal C-reactive protein levels for myocardial salvage in patients with acute myocardial infarction is dependent on the type of reperfusion treatment. Eur Heart J.

[26] Rott D, Leibowitz D (2007). STEMI and NSTEMI are two distinct pathophysiological entities. Eur Heart J.

[27] Munir TA, Afzal NM, Rehman H, Ahmed R (2009). C-reactive protein and acute coronary syndrome: correlation with traditional risk factors, diagnostic cardiac biomarkers, and ejection fraction. Rawal Med J.

